# Terson’s Syndrome with Roth Spot-Resembling Features and Third Nerve Palsy without Radiologically Diagnosed Subarachnoid Haemorrhage

**DOI:** 10.3390/vision8040061

**Published:** 2024-10-07

**Authors:** Olga E. Makri, Iasonas K. Tsekouras, Stylianos N. Mastronikolis, Vasileios E. Panagiotopoulos, Constantine Constantoyannis, Constantinos D. Georgakopoulos

**Affiliations:** 1 Department of Ophthalmology, Medical School, University of Patras, 26504 Patras, Greece; makriolga@upatras.gr (O.E.M.); iktsek@yahoo.gr (I.K.T.); mastronikst@gmail.com (S.N.M.); 2 Department of Neurosurgery, Medical School, University of Patras, 26504 Patras, Greece; baspan@upatras.gr (V.E.P.); cconst@upatras.gr (C.C.)

**Keywords:** Terson’s syndrome, Roth spots, third nerve palsy, posterior communicating artery aneurysm

## Abstract

We report an unusual case of pupil-involving third nerve palsy associated with Terson’s syndrome that resulted in the diagnosis of a right posterior communicating artery aneurysm. Interestingly, Terson’s syndrome presented with Roth spot-resembling features, accompanied by third nerve palsy in a patient without any disturbance of consciousness. To our knowledge, the association of Terson’s syndrome with third nerve palsy has not been described before in the absence of radiologically diagnosed subarachnoid haemorrhage. We present the case of a 48-year-old woman who presented in the Department of Emergencies of the University Hospital of Patras with right-sided complete-pupil-involving third nerve palsy combined with bilateral Terson’s syndrome. More precisely, fundoscopy revealed multiple scattered intra- and pre-retinal haemorrhages in both eyes, while some retinal haemorrhages had a white-centred pattern resembling Roth spots. Neurological examination revealed no nuchal rigidity or any other neurological signs. The Glasgow coma scale score was 15/15. A cerebral computed tomography angiography revealed a 5 mm aneurysm of the right posterior communicating artery and the patient underwent a microvascular clipping operation after craniotomy. The significant contribution of the ophthalmological consultation to the appropriate and timely diagnosis and management of a life-threatening condition is highlighted.

## 1. Introduction

Terson’s syndrome is defined as the presence of vitreous, preretinal, intraretinal or sub-retinal haemorrhage in the setting of subarachnoid bleeding or as increased intracranial pressure after traumatic or non-traumatic intracerebral haemorrhage or even after traumatic brain injury with no intracranial bleeding [1,2]. The most common association of Terson’s syndrome is considered to be subarachnoid haemorrhage. The occurrence of Terson’s syndrome in cases of subarachnoid haemorrhage results in a worse prognosis and significantly increased mortality [3,4]. Terson’s syndrome is usually associated with coma, a Glasgow coma scale score of less than eight or with severe neurological findings [5].

Acquired third nerve palsies are of particular importance since they might be caused by life-threatening intracranial aneurysms [5]. In complete oculomotor nerve palsy, the eye is positioned downwards and outwards, and there is also complete ptosis and a dilated pupil.A pupil-involving third nerve palsy comprises a medical emergency due to the possibility of the presence of an enlarging intracranial aneurysm more frequently at the posterior communicating artery [6]. A pupil-sparing third nerve palsy also warrants extensive investigation, as internal ophthalmoplegia may also develop later during the course of the disease. However, pupil-sparing third nerve palsies due to an intracranial aneurysm have also been reported [7].

We describe a case of Terson’s syndrome with Roth spot-resembling features, accompanied by a pupil-involving third nerve palsy in a patient without any radiologically diagnosed subarachnoid haemorrhage or disturbance of consciousness that resulted in the diagnosis of a right posterior communicating artery aneurysm.

## 2. Detailed Case Description

A 48-year-old Caucasian female was examined at the Emergency Department of the University Hospital of Patras. The patient presented with a 9-day history of ptosis of her right eyelid followed by a sudden decrease in vision of her left eye (OS). Her previous ocular and medical history were unremarkable.

When asked, the patient mentioned an episode of short-duration intense headache combined with a single vomit 11 days ago. Neurological examination revealed a pupil-involving third nerve palsy without nuchal rigidity or any other neurological signs. The Glasgow coma scale score was 15/15.

**Figure 1:**
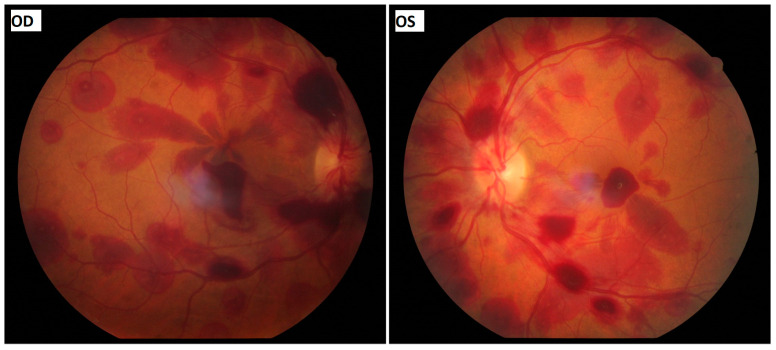
Colour fundus images showing multiple scattered intra- and pre-retinal haemorrhages in both eyes. (OD: right eye, OS: left eye).

An ophthalmological examination confirmed the diagnosis of complete-pupil-involving oculomotor nerve palsy, as there was ptosis of the patient’s right eyelid, the right eye (OD) was positioned downwards and outwards with inability to adduct, supraduct and infraduct and the pupil of the OD was fixed at 5.5 mm in diameter. The direct and consensual pupillary reflexes of the OS were normal, and the diameter of the pupil was 3 mm in photopic conditions. The patient’s best corrected visual acuity was 20/100 in the OD and 20/70 in the OS. The anterior segment examination was unremarkable. Dilated fundoscopy revealed multiple scattered intra- and pre-retinal haemorrhages in both eyes (Figure 1). Several retinal haemorrhages in both eyes had a white-centred pattern resembling Roth spots.Subtle optic disc oedema was observed, especially in the OD. These findings were compatible with bilateral Terson’s syndrome. Although Roth spot-resembling features suggested the possibility of underlying haematological disease, the presence of a pupil-involving third nerve palsy constituted a neurosurgical emergency.

**Figure 2:**
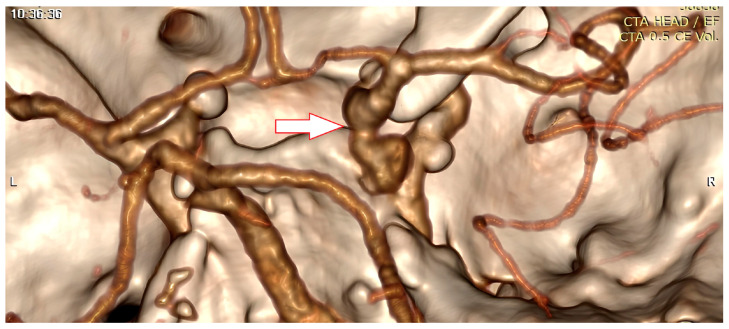
CT angiography image of a 5 mm aneurysm (arrow) of the right posterior communicating artery.

A cerebral computed tomography angiography revealed a 5 mm aneurysm of the right posterior communicating artery (Figure 2). A lumbar puncture was also performed. The opening pressure was normal and the red blood cells count in the cerebrospinal fluid was 678 cells/mm^3^. The patient underwent a right pterional craniotomy and subsequent clipping of the aneurysm that compressed the right oculomotor nerve. No subarachnoid haemorrhage has been documented intraoperatively.

A post-operative ophthalmological examination three months after the surgery showed full absorption of the intra- and pre-retinal haemorrhages in both eyes. The patient’s best corrected visual acuity at that point was 20/30 in the OD and 20/20 in the OS.

## 3. Discussion

Terson’s syndrome, although under-reported, seems to develop in almost 40% of cases with acute aneurysmal intracranial bleeds [8]. It can be bilateral or unilateral and is characterised by the presence of any type of intraocular haemorrhage involving several retinal layers. Vitreous haemorrhage is the most common type of intraocular haemorrhage in Terson’s syndrome and results in a significant decrease in visual acuity [9]. Several possible pathogenetic mechanisms for Terson’s syndrome have been proposed but the debate remains. The most accepted theory suggests an acute, even transient, spike in the intracranial pressure, usually due to an aneurysmal rupture, that leads to increased hydrostatic pressure in the ocular venous system, a decrease in venous return, venous stasis and the rupture of thin superficial retinal vessels [10]. Terson’s syndrome is usually associated with coma, a Glasgow coma scale score of less than eight or with severe neurological findings [8]. Nevertheless, there have been two published cases where Terson’s syndrome occurred without any neurological signs [11,12].

The presence of Roth spot-resembling features in our case suggested the possibility of underlying haematological disease. The presence, however, of a pupil-involving third nerve palsy constituted a neurosurgical emergency, due to the possibility of the presence of an enlarging intracranial aneurysm.

The association of Terson’s syndrome with a pupil-involving third nerve palsy is well-known and has been reported before but, to our knowledge, only in the setting of radiologically diagnosed subarachnoid haemorrhage [13]. In our case, there were no radiological or intraoperative indications of subarachnoid haemorrhage; there was a normal opening pressure at lumbar puncture and the neuroimaging showed no signs of increased intracranial pressure. Only the red blood cell count in the cerebrospinal fluid was indicative of subarachnoid haemorrhage, since it might have resulted from a minor leak of the aneurysm some days before [9], with an abrupt, transient increase in intracranial pressure which may have contributed to the pathogenesis of Terson’s syndrome [9]. The above hypothesis, which is consistent with the patient’s history of headache and vomit 2 days prior to the onset of the third nerve palsy, has led us to use the term “Terson’s syndrome” to describe the condition, even in the absence of radiologically diagnosed subarachnoid haemorrhage.

## 4. Conclusions

We presented a case of pupil-involving third nerve palsy associated with Terson’s syndrome, in which the treatment priority was the management of the aneurysm before a possible major rupture. Our case emphasises that the ophthalmological consultation may assist the diagnosis of a life-threatening condition, as an oculomotor nerve palsy, either pupil-involving or pupil-sparing, warrants investigation for a possible enlarging intracranial aneurysm and Terson’s syndrome may additionally be indicative of minor cerebral aneurysm rupture. A high index of suspicion is required in order to recognise even the less typical cases.

## Data Availability

No new data were created.
